# NK Count and Natural Cytotoxicity in Immune Nonresponders Versus Responders Living With HIV

**DOI:** 10.1002/jmv.70170

**Published:** 2025-01-27

**Authors:** Charlotte Silvestre, Edouard Tuaillon, Maël‐Morvan Duroyon, Magali Abrantes, Jenny‐Constanza Thibout, Martin Villalba, Marie‐Christine Picot, Antoine Gross, Alain Makinson

**Affiliations:** ^1^ IRIM, Université de Montpellier, CNRS Montpellier France; ^2^ Virology Department, University Hospital Montpellier & Inserm U1058 University Montpellier Montpellier France; ^3^ Epidemiology and Clinical Research Unit University Hospital Montpellier Montpellier France; ^4^ Infectious Diseases Department University Hospital Montpellier Montpellier France; ^5^ IRMB, INSERM, CNRS, University Hospital Montpellier University Montpellier Montpellier France; ^6^ Infectious Diseases Department, University Hospital Montpellier & INSERM U1175 University Montpellier Montpellier France

**Keywords:** cytotoxicity, HIV, immune nonresponse, natural killer cells

## Abstract

Despite viral suppression with antiretroviral therapy, immune nonresponders (INR) among people living with HIV (PLWH) still have a higher risk of developing AIDS‐related and non‐AIDS‐related complications. Our study aimed to investigate the phenotype and functions of Natural Killer (NK) cells in INR, to better understand underlying mechanisms of immune nonresponse. Our cross‐sectional study included PLWH aged over 45 with an undetectable HIV viral load sustained for at least 2 years. Participant CD4^+^ T‐cell counts exceeded 500 cells/mm^3^ for IR or below 350 cells/mm^3^ for INR. We characterized NK cell subsets, performed natural cytotoxicity and ADCC assays, and phenotyped NK cells before and after functional assays. Median CD4 levels were 247.5 (IQR, 208.8–286.3) cells/mm^3^ and 768.5 (IQR, 673.3–957.8) cells/mm^3^ in the INR (*n* = 20) and IR (*n* = 40), respectively. NK cell counts were lower in INR (*p* = 0.00066), but the percentages were similar between IR and INR. NKG2A was the only differentially expressed marker between groups, showing higher expression in INR. Additionally, NK cell natural cytotoxicity was elevated in the INR group. Multivariate analyses associated CD4 nadir and low NK cell count with immune nonresponse. Our results do not indicate a clear role for NK‐mediated ADCC in immune nonresponse. We did not objectify an association between CD8 hyperactivation and INR.

## 1 Introduction

People living with HIV (PLWH) are expected to achieve an undetectable HIV viral load under antiretroviral therapy (ART) to restore CD4^+^ T‐cell levels. However, 10%–40% of treated PLWH fail to achieve immune reconstitution despite viral suppression [1, 2, 3, 4, 5]. These immune nonresponders (INR) are at increased risk of developing AIDS‐related and non‐AIDS‐related comorbidities, such as cardiovascular and neurological diseases, cancers, and death [1, 6, 7, 8, 9, 10]. Factors associated with INR include older age [11, 12, 13], a low CD4 nadir [14], a diminished CD4/CD8 ratio at baseline [15], and suboptimal virological response following initiation of ART [16].

The mechanisms behind immune nonresponse remain insufficiently understood [17]. Hypotheses include impaired production or maturation of CD4 cells and chronic immune activation. Microbial translocation, gut impairment, and coinfections with hepatitis B virus (HBV) [18], hepatitis C virus (HCV) [19], or cytomegalovirus (hCMV) [11, 20] may contribute to an altered inflammatory environment even among PLWH receiving ART. Immune senescence may also play an active role in INR.

Natural killer (NK) cells, a subset of innate lymphoid cells, play a crucial role in eliminating stressed, tumor, or infected cells. They exert their cytotoxic activity through direct cell‐mediated cytotoxicity and via antibody‐dependent cellular cytotoxicity (ADCC), wherein antibodies allow infected cell recognition [21]. NK cells are a heterogeneous cell type composed of various subpopulations distinguished by their receptors, maturation states, and functions. With effective ART and CD4 T‐cell restoration, NK cells CD56 and CD16 expression normalized though other markers remained dysregulated [22, 23]. Upon chronic HIV and/or hCMV infection, NK cells exhibit increased maturation, characterized by higher levels of CD57 and NKG2C expression [24]. They also display a more exhausted phenotype, indicated by elevated expression of exhaustion markers such as PD1, Tim3, and TIGIT [25].

NK cells have been investigated in INR, but most studies have primarily focused on describing NK subsets and the phenotypes of NK cells [26, 27, 28]. Few studies have explored mechanisms involving natural cytotoxicity and ADCC in NK cells that might contribute to CD4 T‐cell depletion [29, 30, 31]. We aimed to study the differences in terms of NK cell phenotypes and functions in INR and IR in a well‐matched study.

## 2 Materials and Methods

### 2.1 Ethics Approval

The INKASE study was approved by the Ethics Committee Sud‐Est 1, France (study no. 2021‐A02470‐41), registered in ClinicalTrials.gov (Identifier: NCT05243381) and complied with the Helsinki Declaration. All volunteers gave informed consent.

### 2.2 Participant Enrolment

The study was conducted at the Infectious and Tropical Diseases Department of Montpellier Hospital University. PLWH were proposed to participate in the study if they fulfilled the following criteria: age over 45 years, sustained undetectable HIV viral load for at least 2 years, and at least two CD4^+^ T‐cell count measurements within the past 2 years. IR were defined as individuals with a CD4 lymphocyte count exceeding 500 cells/mm^3^, and INR as individuals with a CD4 lymphocyte count below 350 cells/mm^3^, both groups fulfilling all other inclusion criteria. Noninclusion criteria were an absence of ART, immunosuppressive comedication, ongoing or history of cancer within 5 years of inclusion, pregnancy or breastfeeding, adult protection by law, or absence of social security. Immunosuppressive medication and a history of cancer during the last 5 years were considered as specific conditions of INR and NK disturbances, and PLWH with these conditions were excluded.

We planned to include 20 INR PLWH matched on age (per 5‐year classes), gender, and duration of HIV undetectability (2–8 years and > 8 years) with 40 PLW with IR. After obtaining informed consent, blood samples of 20 mL in EDTA tubes were collected from each volunteer.

### 2.3 Isolation of NK Cells in PBMC

All samples were processed within 4 h of blood collection. After PBMC isolation by density gradient, the percentage of NK cells was determined as live cells within PBMC negative for lineage markers (CD3^neg^ CD4^neg^ CD14^neg^ CD19^neg^) and positive for CD56 and CD16 markers.

NK cells were isolated using the human NK cell isolation kit (Miltenyi Biotec) following the manufacturer's protocol. Isolated NK cells and plasmas were stored at −80°C for further analysis.

### 2.4 NK Cell Phenotype

Isolated NK cells from the cohort were thawed and phenotyped with two antibody panels (Table S1) and a viability marker (FVS510, BD Biosciences). The panels shared a backbone composed of a negative lineage (CD3‐FITC, CD4‐FITC, CD14‐FITC, and CD19‐FITC), CD56‐BV786, CD16‐PerCP‐Vio700, CD57‐BV605, NKG2C‐PE‐Vio770, NKG2D‐BV711, NKp30‐PE, Tim3‐PE‐Cy5, and CD107a‐BV‐421. NKG2A‐APC‐VioBrightR720 and IFNγ‐APC were added to Panel 1, whereas NKp46‐APC‐VioBrightR720 and CD3ζ‐APC were added to Panel 2. After performing functional assays (natural cytotoxicity and ADCC assays), NK cell phenotypes were assessed with an effector‐to‐target (E/T) ratio of 1:1.

### 2.5 Flow Cytometry Data Acquisition and Analysis

The data were acquired on a BD LSR Fortessa cytometer. Data was firstly cleaned with FlowJo v10.10 using FlowAI plugin (the gating strategy is presented in Figure S5). Then, calculations, representations, and statistics were done using R v4.3.2 (https://www.R-project.org/) with the Bioconductor packages Catalyst 1.28.0 (https://github.com/HelenaLC/CATALYST) and diffcyt 1.24.0 (Weber (2024), https://github.com/lmweber/diffcyt). All cytometry data from both panels and participants, including phenotyping after functional tests, are included in FlowSOM clusterization. After a first clusterization with 100 clusters based on CD16, CD56, CD57, NKG2C, NKG2D, and NKp30 expressions, an 8‐metaclusterization was performed according to the delta area graph depicted in Figure S4 and package instructions. The Uniform Manifold Approximation and Projection (UMAP) was used to represent a dimensional reduction of NK cells. For subsequent analyses, the single‐cell experiment object produced by CATALYST was parsed as required. We established three levels of expression for the different markers, based on the tertiles of each marker: high expression (high, expression: > 66%), intermediate level (int, expression: 33%–66%), and low expression (low, expression: 0%–33%).

### 2.6 Natural Cytotoxicity Assay

To evaluate the natural cytotoxicity of NK cells, target K562 cells [32] were stained with Carboxyfluorescein succinimidyl ester (CFSE, Thermofisher) and cocultured with NK cells at different ratios (E/T: 1/1; 0.5/1; 0.25/1; 0.125/1) during 5 h at 37°C. Cell mortality was evaluated after staining with FVS700 (BD Biosciences) by determining the percentage of CFSE^pos^FVS700^pos^ cell in CFSE^pos^ cells on a Novocyte cytometer and Novoexpress software (Agilent). For each individual, the natural cytotoxicity index was computed as follows: $\mathrm{Index}=\max\left(\frac{\%\mathrm{of deadcells}}{E/T\mathrm{ratio}}\right)$.

### 2.7 ADCC Assay

To evaluate ADCC, CEM.NKR‐CCR5 [33] (NIH AIDS Reagent Program) cells were stained with CFSE. The labeled cells were incubated with a dose of 0.33 µg/mL of recombinant GP120 Bal (HRP_20082, NIH AIDS Reagent Program) as a virological target before being incubated with serum. The plasma of PLWH or referenced antibodies HIVIG (ARP‐3957, NIH AIDS Reagent Program) were added before the coculture with NK cells at different ratios (E/T: 1/1; 0.5/1; 0.25/1; 0.125/1) for 5 h at 37°C. Cell mortality was evaluated after staining with FVS700 by determining the percentage of CFSE^pos^FVS700^pos^ cells in CFSE^pos^ cells on a Novocyte cytometer. The ADCC index was computed as follows: $\mathrm{ADCC}\;\mathrm{Index}=\max\left(\frac{\%\mathrm{of}\;\mathrm{dead}\;\mathrm{cells}\;\times \;\mathrm{antibody}\;\mathrm{dilution}}{E/T\mathrm{ratio}}\right)$. The normalized ADCC index was computed as follows: $\mathrm{Normalized}\mathrm{ADCC}\;\mathrm{Index}=\frac{\mathrm{ADCC}\;\mathrm{Index}\;\mathrm{with}\;\mathrm{PLWH}\;\mathrm{plasma}}{\mathrm{ADCC}\;\mathrm{Index}\;\mathrm{with}\;\mathrm{reference}\;\mathrm{antibody}}$.

### 2.8 Data Collection

Biological data, including CD4 cell count and percentage, CD8 cell count and percentage, CD4/CD8 ratio, and CD4 nadir, were collected. Clinical data, such as history of AIDS and coinfections with hCMV, HBV, or HCV, were also recorded. Coinfections with hCMV were defined based on the presence of anti‐CMV antibodies, while HBV and HCV coinfections were defined by the presence of HBs antigen and anti‐HCV antibodies, respectively.

### 2.9 Statistical Analysis

All statistical analyses were performed using R (version 4.3.1), with a significance level set at *p* < 0.05. The tests performed in accordance with their conditions of application are indicated in table and figure legends. The adjusted *p* values were computed with Holm's method. The analyses with CATALYST/diffcyt package used diffcyt/edgeR method for cluster abundance and diffcyt/limma method for marker expression, both with significance for fdr < 0.05. The association of parameters with immune nonresponse is analyzed using multivariable logistic regression with glm function.

## 3 Results

### 3.1 Clinical Characteristics of Enrolled Participants

The matching criteria of age, proportion of men, and duration of HIV aviremia were similar in both groups (Table 1). Median CD4 levels were 247.5 (IQR, 208.8–286.3) and 768.5 (IQR, 673.3–957.8) cells/mm^3^ in the INR group and IR group, respectively (Table 1). History of AIDS was significantly more frequent in INR (55% vs. 27.5%). Coinfection with CMV, HCV, or HBV did not differ between groups. Percentage of CD4 counts, absolute count, and CD4/CD8 ratio were significantly lower in INR than in IR.

**Table 1:** Characteristics of PLWH included in the study. INR and IR groups were matched on age, gender, and duration of HIV aviremia.

	INR group (*n* = 20)	IR group (*n* = 40)	*p* value
Age (years)	64 [56.5–69.75]	63.5 [54.75–69]	0.951†
Men	17 (85%)	34 (85%)	> 0.999‡
HIV aviremia duration (years)	10.2 [6.1–8.1]	10 [8.1–14]	0.499†
History of AIDS	11/20 (55%)	11/40 (27.5%)	0.037§
Anti‐hCMV antibodies	18/20 (90%)	34/40 (85%)	0.707‡
Anti‐HCV antibodies	3/20 (15%)	3/40 (7.5%)	0.390‡
HBs antigen	3/20 (15%)	4/40 (10%)	0.676‡
CD4 count (cells/mm^3^)	247.5 [208.8–286.3]	768.5 [673.3–957.8]	< 0.001¶
CD4 percentage (%)	23.5 [17.25–29.95]	39.5 [32.63–46.03]	< 0.001†
CD8 count (cells/mm^3^)	420 [318.8–614.5]	772 [474.3–1028]	0.011¶
CD8 percentage (%)	42.2 [34.03–49]	33.7 [26.85–41.8]	0.009†
CD4/CD8 ratio	0.585 [0.4–0.695]	1.1 [0.8–1.675]	< 0.001¶
CD4 nadir (cells/mm^3^)	35.09 [24.25–106.6]	287.5 [106.3–399]	< 0.001¤

*Note:* The values presented for continuous variables are median and IQR.

^†^ Two‐sample *t*‐test.

^‡^ Fisher's exact test.

^§^ Pearson's χ^2^ test.

^¶^ Wilcoxon rank‐sum test.

^¤^ Wilcoxon rank‐sum exact test.

### 3.2 NK Cell Counts

**Figure 1:**
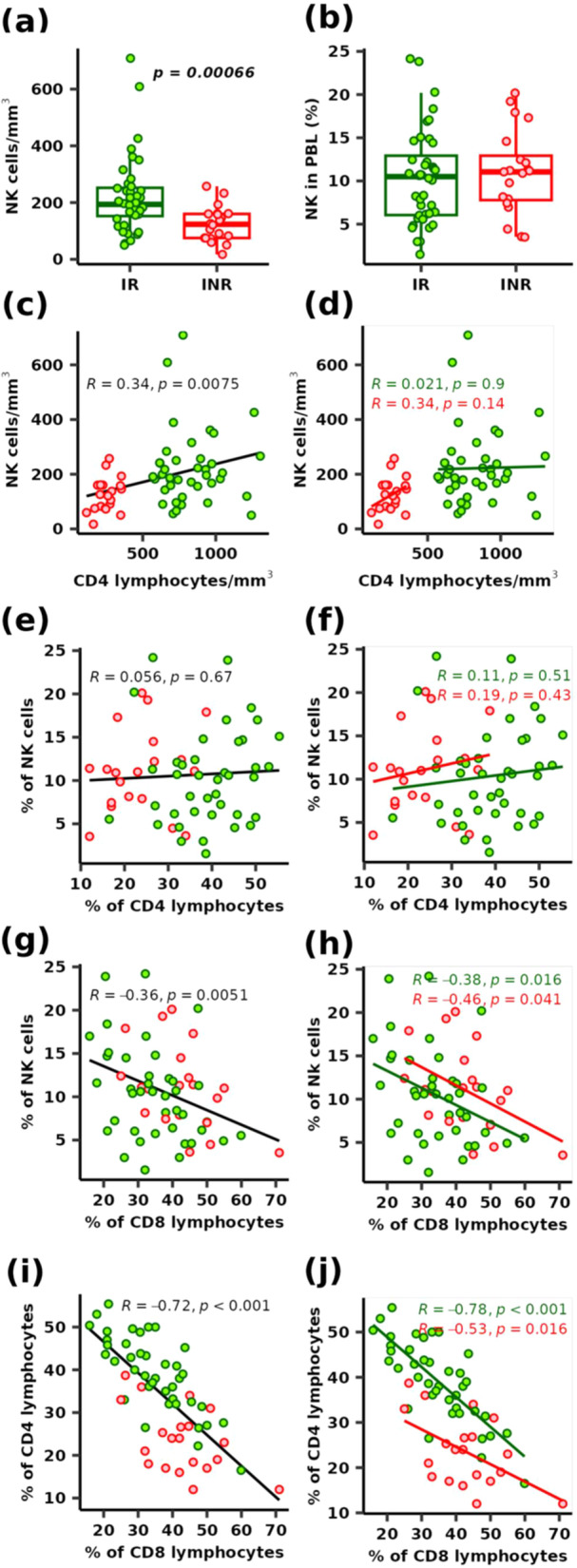
The estimated number and percentage of natural killer (NK) cells. The estimated number (a) and percentage of NK cells among total leukocytes (b) in both the IR (green) and the INR (red) groups. The significant *p* value of the Wilcoxon rank‐sum test is indicated on the graph. Correlation between the estimated number of NK cells and CD4 count in all enrolled individuals (c) and within each group (d). Correlation between NK cell percentage and CD4 percentage in all enrolled individuals (e) and within each group (f). Correlation between NK cell percentage and CD8 percentage in all enrolled individuals (g) and within each group (h). Correlation between CD8 cell percentage and CD4 percentage in all enrolled individuals (i) and within each group (j). Pearson's correlation estimates and *p* values are indicated on graphs.

There was a significantly lower count of NK cells in INR compared to IR (*p* = 0.00066), but no significant differences in percentages (*p* = 0.52). The median NK cell counts and percentages were 124 cells/mm^3^ (IQR, 75.1–160) and 11.1% of total leukocytes, respectively, in INR and 194 cells/mm^3^ (IQR, 153–252) and 10.5% of total leukocytes in the IR (Figure 1a,b). The age distribution analysis (Figure S1a) did not reveal a significant depletion trend of NK cells with increasing age in IR and INR. NK cell counts correlated with CD4 T‐cell counts (*R* = 0.34, *p* = 0.0075) in all volunteers (Figure 1c), but not in groups of IR and INR, despite a trend in INR (Figure 1d). The percentage of NK cells (Figure 1e,f) relative to the percentage of CD4 cells remained stable globally and in each group. In contrast, the percentages of NK and CD4 cells were inversely correlated with CD8 percentages (Figure 1g,j) in all volunteers (*R* = −0.72, *p*< 0.0001 and *R* = −0.36, *p*= 0.0051, respectively) and within each group. There was a positive correlation between NK and CD4 cell counts and CD8 cell counts (Figure S1b–e). There was also no difference in the percentage of hyperactivated CD8 lymphocytes (CD38^+^CD8^+^) between the two groups (Figure S1f).

### 3.3 Expressions of NK Cell Markers

**Figure 2:**
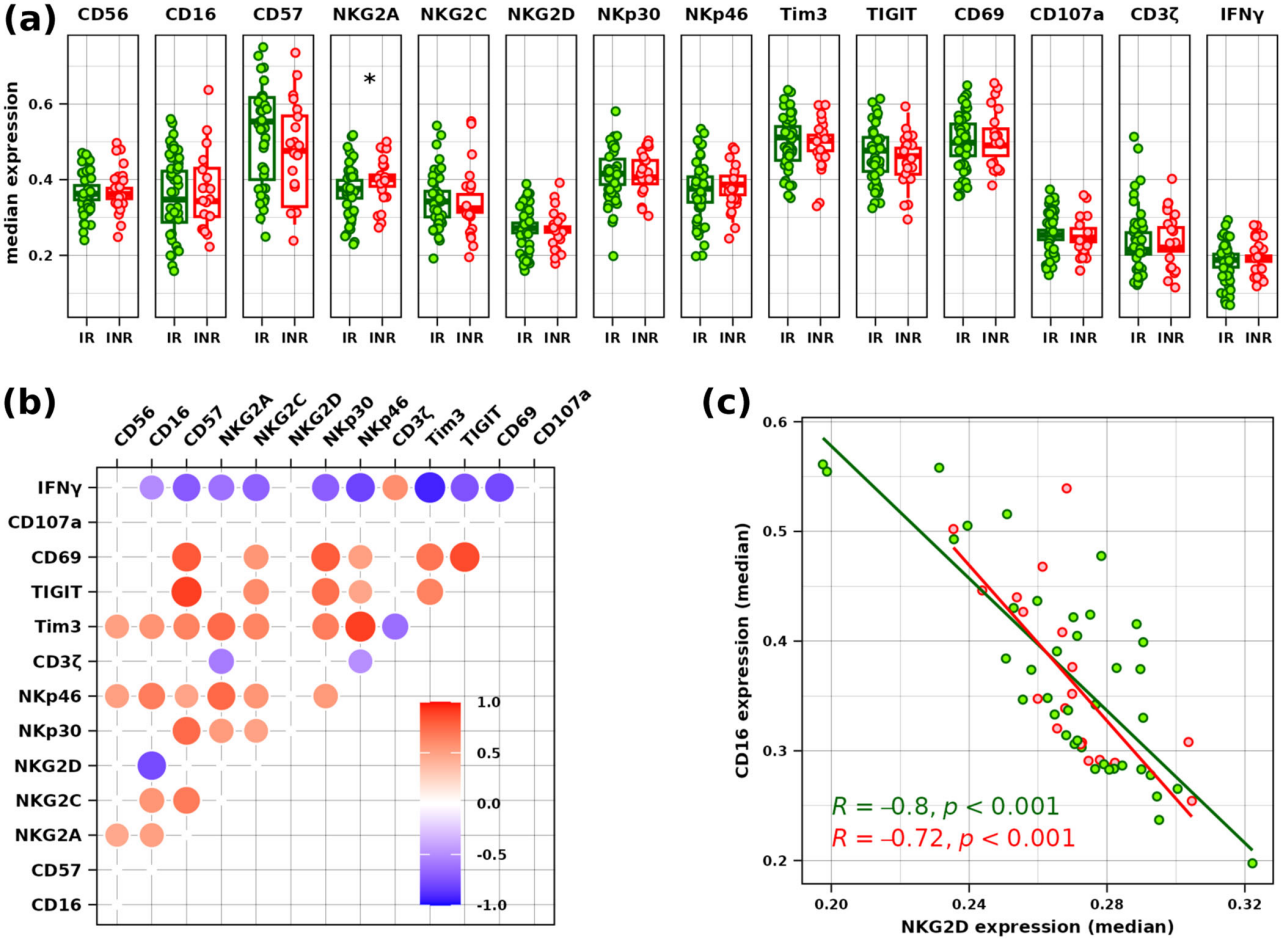
Expression level of all markers in the IR and INR groups. Comparison of marker expression measured by flow cytometry in both IR (green) and INR (red) groups (a). * indicate Wilcoxon rank‐sum test adjusted *p* < 0.05. (b) Correlation matrix plot of marker expression. Significant correlations (Pearson's test adjusted *p* < 0.05) are indicated by circles, with color and size indicating correlation estimate levels. (c) Highlights of the negative correlation between CD16 and NKG2D levels in both IR and INR groups.

Expression levels of phenotypic NK markers showed differences in INR and IR groups in the expression of CD57 and NKG2A (Figure 2a). NKG2A was significantly higher in INR (adjusted *p* = 0.042). The immune checkpoint markers Tim3 and TIGIT, as well as the activation marker CD69, were similarly highly expressed in both groups. Correlations between the expression levels of phenotypic markers are depicted in Figure 2b. Positive correlations included TIGIT and CD57, CD69 and TIGIT, Tim3 and NKp46, CD57 and NKp30, and NKG2A and NKp46. Conversely, the most notable negative correlations were CD16 and NKG2D, IFNγ and NKp46, and IFNγ and Tim3. The negative correlation between CD16 and NKG2D is illustrated in Figure 2c. Both INR and IR cells exhibited similar correlation factors (*R* = −0.72 and −0.80, respectively).

### 3.4 NK Cell Clustering

**Figure 3:**
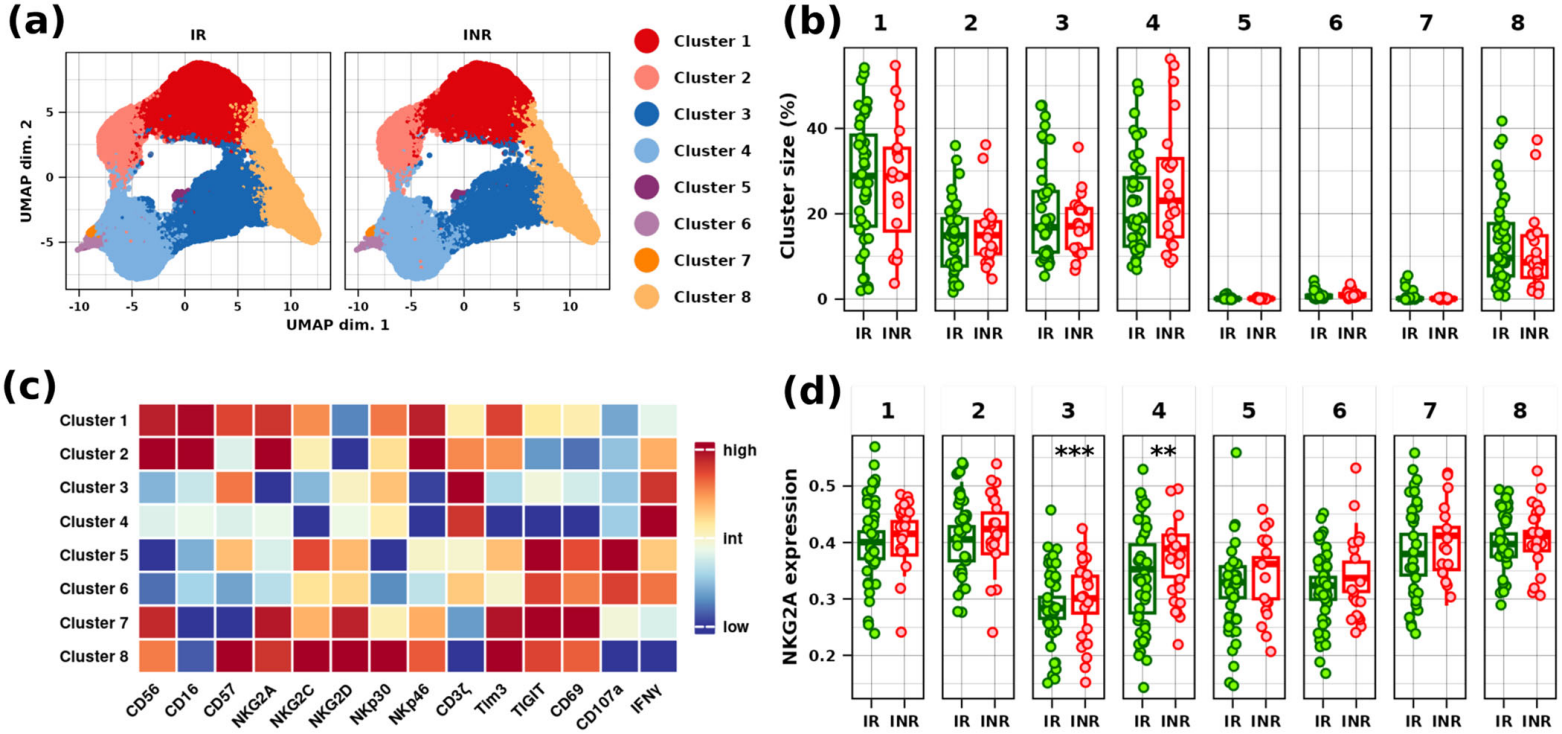
Clusterization and expression levels of markers in the clusters. (a) Uniform Manifold Approximation and Projection (UMAP) representation of the 8‐metaclusterization. (b) Frequencies of the clusters in the IR (green) and INR (red) groups. (c) Expression heatmap of all markers across the eight clusters in all individuals. (d) NKG2A expression levels considering clusters by groups. Statistically significant intergroup differences are indicated on graphs (*** for adjusted *p* value < 0.01 and ** for adjusted *p* value < 0.05, obtained after diffcyt/limma analysis).

Figure 3a shows the UMAP graphs resulting from NK cell metaclustering from all INKASE participants, categorized by groups. The abundance of eight clusters is evaluated (Figure 3b), with Clusters 1, 2, 3, 4, and 8 being the most abundant. Clusters 5, 6, and 7 collectively represent only 1.5% of total NK cells. Diffcyt/EdgeR analysis did not detect differences in cluster abundances between INR/IR groups. Figure 3c depicts a heatmap of marker expression levels (for UMAP graph projections of each marker expression, see Figure S2). Clusters 1 and 2 express high levels of CD16, which were associated with heightened cytotoxicity subsets. They shared many markers but can be differentiated by the CD57 expression level, which is higher in Cluster 1. Clusters 3 and 4 expressed intermediate levels of CD16 and high levels of CD3ζ and IFNγ. These clusters were differentiated by their level of maturation, with Cluster 3 showing higher expression of CD57. Cluster 8 exhibited high expression levels of most markers, except for CD16, CD3ζ, CD107a, and IFNγ, and so could have poor functional activity. Marker expression levels in clusters were compared between the two groups with diffcyt package. Among markers, NKG2A expression is consistently elevated among INR, with significant differences in Clusters 3 and 4 (Figure 3d).

### 3.5 Natural Cytotoxicity and ADCC

**Figure 4:**
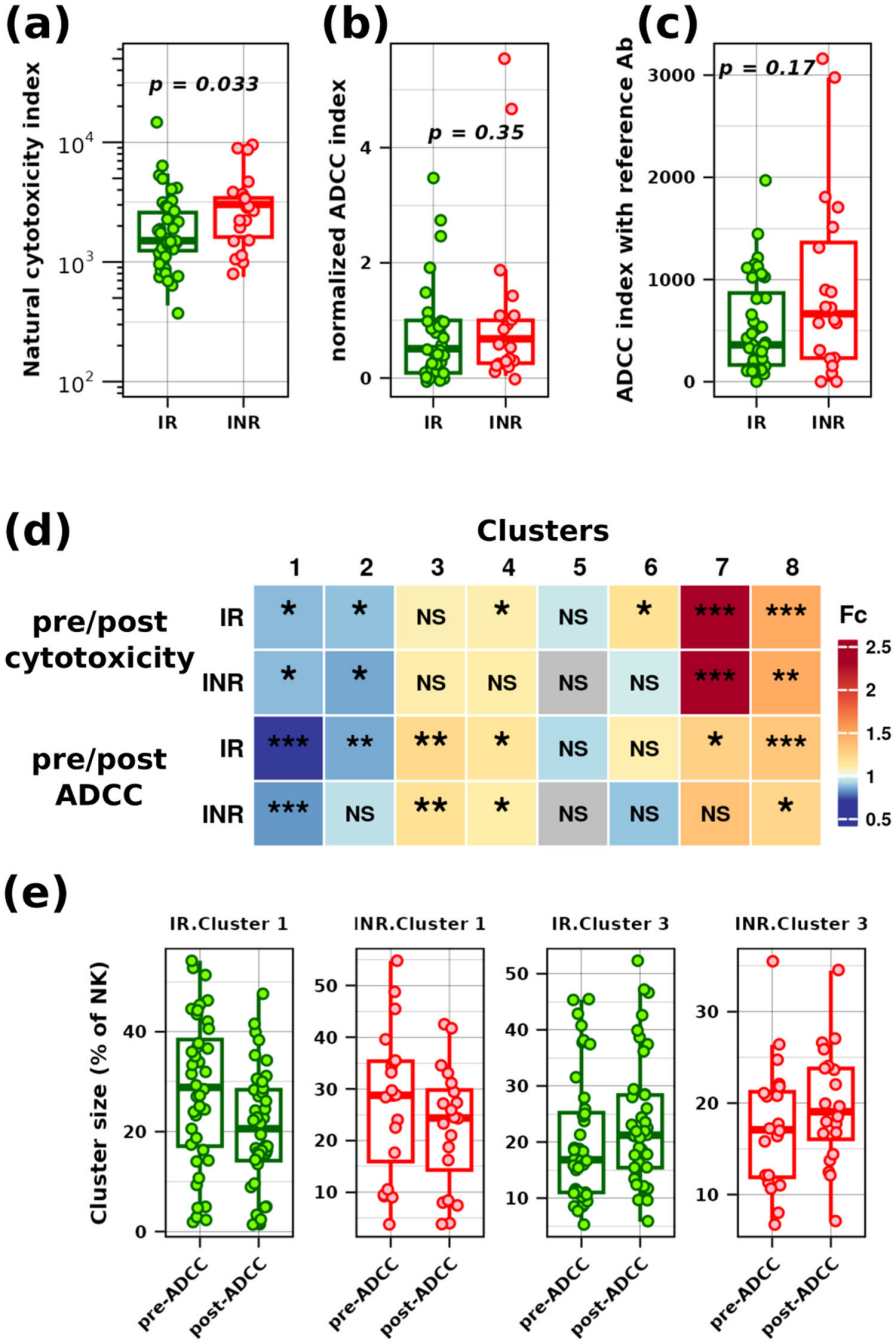
Natural cytotoxicity and ADCC functional assays in the IR and INR groups. Natural cytotoxicity index (a), normalized ADCC index (b), and referenced HIVIG ADCC index (c) in the IR (green) and INR (red) groups; *p* values from the Wilcoxon rank‐sum test are indicated on graphs. (d) and (e): Variations in cluster abundance before and after natural cytotoxicity and ADCC assays are presented on heatmap (d) with a focus on Clusters 1 and 3 (e). Heatmap colors indicated abundance fold changes; significances computed by diffcyt/EdgeR method are indicated by NS (adjusted *p* value > 0.05), * (adjusted *p* value < 0.05), ** (adjusted *p* value < 0.01), and *** (adjusted *p* value < 0.001).

The natural cytotoxicity activity of NK cells was higher in INR than IR (*p* = 0.033), as illustrated in Figure 4a. No significant differences were observed in the ADCC index with autologous plasma normalized to HIVIG between IR and INR groups (Figure 4b, *p*= 0.17) or in the ADCC index using the referenced antibody‐pool HIVIG (Figure 4c, *p* = 0.35).

Figure 4d presents a heatmap comparing the abundance of clusters in both INR and IR groups before and after natural cytotoxicity assays (pre/post‐cytotoxicity) and ADCC assays (pre/post‐ADCC) obtained through diffcyt/edgeR analysis. Clusters 1 and 2, expressing high levels of CD16, decreased after the functional assays, except for Cluster 2 in the INR group pre‐ versus post‐ADCC comparison. Clusters 3 and 4, characterized by an intermediate level of CD16, were slightly enriched after ADCC assays and after natural cytotoxicity for the IR group. Clusters 7 and 8 were enriched after the functional assays, mainly after natural cytotoxicity activity with the exception of Cluster 7, which remained unchanged in the ADCC assay for the INR group. Figure 4e illustrates the variations showing slight depletion of Cluster 1 and concomitant enrichment of Cluster 3 in both groups.

The variations in marker expressions before and after natural cytotoxicity and ADCC assays are respectively presented in Figure S3.

### 3.6 Association of Factors With Immune Nonresponse

**Figure 5:**
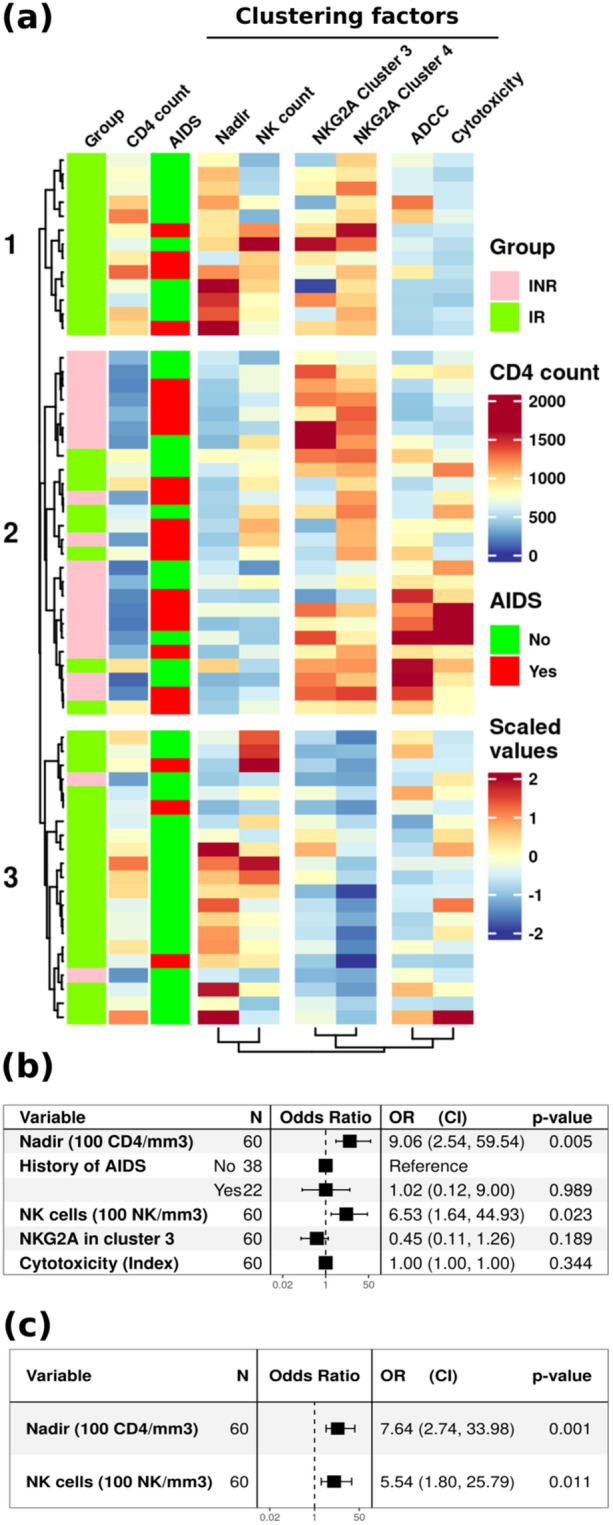
Factors associated with immune nonresponse. (a) Clustering heatmap of previous significant factors associated with immune nonresponse. Each row corresponds to an individual. Left annotations: Group, CD4 count, and AIDS history are not included in clustering. The heatmap is horizontally split regarding dendrogram of individuals (left) and vertically split regarding factor dendrogram (below). Forest plots illustrate the impact of each factor on immune nonresponse status (b), highlighting those associated with immune nonresponse status (c). Odds ratio (OR) and their confidence intervals (CIs) and *p* value obtained with multivariable logistic regression are indicated on graphs.

Figure 5a presents a clustering heatmap illustrating factors of the previous analyses for the 60 PLWH enrolled in INKASE study and annotated the heatmap with group, CD4 count, and AIDS history. The cell counts, represented by CD4 nadir and NK count, clustered together. NKG2A expression in Clusters 3 and 4 grouped together. Functional indices calculated after natural cytotoxicity and ADCC assays formed the third group. The dendrogram of individuals (left of the heatmap) allowed the delineation of three distinct groups among the PLWH. The first group comprised 13 IR with a low natural cytotoxicity index and high NKG2A expression in NK cell Cluster 4. The second group predominantly consisted of INR (18 INR, representing 90% of the INR group) along with 8 IR individuals, characterized by a low CD4 nadir and high NKG2A expression in Clusters 3 and/or 4. The last group included 2 INR and 19 IR with low NKG2A expression in Clusters 3 and 4. The majority of the INR (18/20) were part of Group 2 related to a history of AIDS, known as an associated factor of immune nonresponse.

The forest plot of Figure 5b shows the association assessment of selected factors (excluding correlated parameters). Among the factors, only CD4 nadir and NK cell count were significantly associated with immune nonresponse. Refining the analysis (Figure 5c) shows that a CD4 nadir variation of 100 cells/mm^3^ is associated with an odds ratio (OR) of 7.64 (CI: 2.74–33.98, *p* = 0.001) and a variation of 100 NK/mm^3^ with an OR of 5.54 (1.80–25.79, *p* = 0011).

## 4 Discussion

We found that in INR, the absolute count of NK was lower than in IR. The percentages of NK were similar in both groups, in contrast with diminished percentages of CD4 T‐cells and increased percentages of CD8 levels in INR. NKG2A expression was elevated in INR, particularly within Clusters 3 and 4 exhibiting an intermediate level of CD16. We also highlighted a higher activity in natural cytotoxicity in INR compared to IR, but not significantly for ADCC. Ultimately, the CD4 nadir and NK cell count were independently associated with immune nonresponse.

The INKASE study stands out as one of the few investigations delving into the phenotype and functions of NK cells, in a well‐matched and translational approach of IR and INR, and compared NK cell phenotypes following functional assays. To explore NK functions, we performed the assays using IL‐2 unstimulated NK cells. This approach allowed us to preserve the basal activity of NK cells despite the thawing process.

There is no consensus definition of INR, and studies have chosen various definitions of INR, based on CD4/CD8 ratios and/or different CD4 cutoffs [1, 15]. However, we chose our cutoffs as CD4 levels < 350 cells/mm^3^ have been linked to AIDS and non‐AIDS‐related comorbidities and malignancies, including Kaposi syndrome, non‐Hodgkin and Hodgkin lymphomas, and lung and liver cancers [34], and have been previously used in immunodiscordant studies [14]. We presumed that excluding PLWH with intermediate levels of CD4 recovery between 351 and 500 cells/mm^3^ would help to clearly distinguish differences in underlying immune mechanisms of INR. We chose not to define INR and IR with the CD4/CD8 ratio, as it can reflect either CD4 non‐restoration (the aim of our study), but also CD8 increase.

The previously known factors associated with immune nonresponse, such as a history of AIDS [35], a low CD4 nadir [14, 35], and a low CD4/CD8 ratio [1, 15, 35], were also observed in our study as expected. Multivariable analyses showed that, in our study, a history of AIDS had no impact on immune nonresponse taking account the nadir. Nevertheless, the two parameters are closely associated since AIDS appears primarily with low CD4 counts.

In addition to nadir, a low NK cell count was significantly associated with immune nonresponse in our study. This has never been formally described before. Interestingly, *Luo* et al. [30] described similar nonsignificant trends, in 13 IR, 11 INR, and 12 healthy controls. IR had an NK cell population at similar levels of healthy controls and INR exhibited a lower NK cell count, as in our study. The sample size and participant homogeneity, ensured by matching criteria, allowed us to characterize the associations in our study.

The INR of INKASE cohort displayed significant decreases in CD8 lymphocyte counts compared to IR (median CD8 count of 420 and 772 cells/mm^3^, respectively). In contrast to decreased CD4 percentage and stable NK cell percentage in INR and IR, CD8 percentages were significantly increased in INR (33.7% in the IR group and 42.2% in the INR group). However, this CD8 increase did not signify increased CD8 activation, as the percentage of activated CD8^+^CD38^+^ lymphocytes was low and similar in both groups. Thus, our study does not highlight CD8 activation as a correlate of INR. Negredo et al. [14] also described a decrease in CD8 counts in INR, but in contrast with our study a decrease in CD8 percentage and a significant increase in CD8 hyperactivation. However, in that same study, CD8 activation remained low between groups (12.4% in INR vs. 8.2% in IR), with differences in values which may not be clinically relevant. Our results need confirmation, and additional studies should determine whether the increased risk of AIDS and non‐AIDS related events objectified in cohorts of INR with low CD4/CD8 [10] is due, in INR, to low CD4 counts or NK counts, rather than CD8 activation, or both.

The clusters representing different NK subtypes showed similar abundance in both groups, indicating that the reduction of NK cell counts is not solely due to the depletion of a specific NK subpopulation. Cluster analysis divided close clusters (Cluster 1 vs. Cluster 2, Cluster 3 vs. Cluster 4) based on CD57 expression levels, with CD57^+^ cells being relatively frequent among participants. This could be linked to the documented increase of CD57^+^ NK cells associated with chronic HIV infection and the high prevalence of CMV infection in the study population [36]. The increase in cytotoxic NK cells (Clusters 1–4) might be enhanced by HIV infection and contribute to higher natural cytotoxicity in INR. However, the abundance of clusters did not differ between the two groups, suggesting that NK cell diversity is not influenced by immune nonresponse and does not participate in it.

NK cells from INR exhibited higher activity in natural cytotoxicity compared to IR. The expression levels of common markers of NK cells were mostly similar between the two groups, except for the inhibitory receptor NKG2A. NKG2A was significantly higher in the INR group, particularly in two clusters within Cluster 3 and Cluster 4, which were characterized by an intermediate level of CD16 and high levels of CD3ζ and IFNγ. NKG2A is an inhibitory receptor of NK activity, and its overexpression could be the result of increased natural NK‐mediated cytotoxicity in INR as the expression of CD16 and NKG2A have been described as opposites [37]. Moreover, NKG2A^+^ NK cells exhibit a higher cytotoxic response against the K562 cells and HIV‐infected CD4 cells [38]. Negredo et al. [14] identified ex vivo CD4 T‐cell death during PBMC culture as predictors of immune nonresponse concurrently to nadir CD4 cells. It would be interesting to determine whether increased NK natural cytotoxicity was implicated in the CD4 death in INR.

There was no significant difference in ADCC function between the two groups when using either autologous plasma antibodies or the referenced HIVIG antibody pool, arguing against a specific mechanism of NK ADCC in immune nonresponse physiology. Indeed, functional activities were associated with cluster variations post‐functional assays in the most abundant clusters but did not differ between INR and IR. However, a recent study has shown that in PLWH with elevated levels of soluble GP120 (sGP120), the CD4^+^ T‐cell count is inversely correlated with the level of anti‐GP120 antibodies targeting the cluster A region [39]. Through its circulation, sGP120 might contribute to CD4^+^ T‐cell depletion via ADCC in vivo. Measuring sGP120 in INR would be relevant and could help anticipate whether Fostemsavir might mitigate the involvement of GP120 and ADCC in INR [40].

We do acknowledge several limitations in our study. First, our study comprised only 20 INR and may not be totally representative of PLWH with INR or IR. In our study, we analyzed individuals several years (3–23 years) after their last detected HIV viremia and do not describe events occurring during (or involved in) the immune nonresponse onset. Second, we did not perform a long‐term follow‐up to determine which parameters are predictive of clinical events, as our study was a cross‐sectional study. Third, in our NK cell functional assays, we used two referenced cell lines, K562 and CEM.NKR‐CCR5. We used cell lines as target cells instead of autologous CD4 T‐cells and identical numbers of NK cells from INR and IR, which may not reflect in vivo NK depletion in INR. These approaches were used to enable comparison of NK functions across all volunteers. Lastly, we characterized NK cells after thawing using two antibody panels, though some NK‐specific markers were lacking. Since no standardized panel exists for NK cell characterization—beyond the commonly used CD56 and CD16 markers, which we used to determine NK counts on PBMCs isolated directly from blood—this made direct comparisons with other studies challenging. As an example, we opted to examine the expression of Tim3 and TIGIT instead of PD‐1 and DNAM‐1. The only inhibitory receptor included was NKG2A, for which we observed a difference between the two groups, and the analysis of KIR receptors and NKp44 and NKp80 would have been important.

The higher natural cytotoxicity in the INR group is associated with increased NKG2A expression in certain NK clusters. Therefore, it would be pertinent to investigate these phenomena alongside markers expressed by CD4 lymphocytes, such as activation markers (HLA‐DR and CD38), cellular exhaustion markers (PD1), the NKG2A‐ligand HLA‐E and its functional interaction with NKG2A, and the expression CMH‐I molecules and other activating ligands for NK cells [28, 35, 41, 42]. This would be valuable for better understanding the relationship between these two cell populations, specifically whether NKG2A expression could confer CD4 lymphocyte resistance to increased NK activity or by opposition that NK ligand expression would have a deleterious on CD4 lymphocyte survival and if the heightened NK cytotoxicity is influenced by CD4 lymphocyte.

In conclusion, in this well‐matched study of INR and IR, we found that NK cells were less abundant but had similar percentages in INR and exhibited a higher natural cytotoxicity response, but not an increased NK ADCC. We did not objectify an association between CD8 hyperactivation and immune nonresponse. Our results do not highlight a clear role of NK‐mediated ADCC in immune nonresponse and suggest limited efficacy of strategies limiting NK ADCC to restore CD4 cell levels. Our results merit confirmation in other studies of INR.

## Author Contributions

**Charlotte Silvestre:** experimental design, experiment, formal analysis, writing–original draft. **Edouard Tuaillon:** medical investigator, experimental design, review. **Maël‐Morvan Duroyon:** biostatistics. **Magali Abrantes:** experiment. **Jenny‐Constanza Thibout:** methodology. **Martin Villalba:** experimental design. **Marie‐Christine Picot:** methodology, biostatistics. **Antoine Gross:** conceptualization, funding acquisition, experimental design, biostatistics, writing–original draft. **Alain Makinson:** conceptualization, principal medical investigator, funding acquisition, methodology, writing–original draft.

## Ethics Statement

The INKASE study was approved by the Ethics Committee Sud‐Est 1, France (study no. 2021‐A02470‐41), registered in ClinicalTrials.gov (Identifier: NCT05243381) and complied with the Helsinki Declaration.

## Consent

All volunteers gave informed consent.

## Conflicts of Interest

The authors declare no conflicts of interest.

## Supporting information

Supporting information.

**Supplementary Figure 1: (A)** Correlation between age and NK count if IR (green) and INR (red) groups. **(B, C)** Correlation between CD8 counts and NK counts in all enrolled individuals **(B)** and within each group **(C). (D, E)** Correlation between CD8 counts and CD4 counts in all enrolled individuals **(D)** and within each group **(E)**. **(F)** Percentages of CD8^+^CD38^+^ lymphocytes among CD8^+^ cells in both IR (green) and INR (red) groups. Dashed horizontal red line indicates 5% cut‐off. P‐value computed by Wilcoxon's rank sum test is indicated on the graph.

**Supplementary Figure 2:** UMAP graphs depicting marker expression levels in NK cell population in IR and INR.

**Supplementary Figure 3:** Marker expressions before and after cytotoxicity (CX) or ADCC in IR (green circle) and INR (red circle). P‐values of Wilcoxon's signed rank test are indicated on corresponding graphs: ns, p > 0.05, * p < 0.05, ** p < 0.01, *** p < 0.001.

**Supplementary Figure 4:** Delta area graph from CATALYST package for metaclusterization process.

**Supplementary Figure 5: Flow cytometry gating strategy on Flowjo**.

## Data Availability

The authors have nothing to report.
