# Supplementary material for: NK Count and Natural Cytotoxicity in Immune Nonresponders Versus Responders Living With HIV
Source: J Med Virol. 2025 Jan 27;97(2):e70170. doi: 10.1002/jmv.70170 (PMC11771562; doi:10.1002/jmv.70170)
Supplement: Supplementary file 1 — Supporting information. [file JMV-97-e70170-s001.docx]

**Table S1**: Natural Killer cells flow cytometry panels

| **Goal of the markers** | **Markers** | **Fluorochrome** | **Clone** | **Brand** |
| --- | --- | --- | --- | --- |
| Negative lineage | CD3 | FITC | REA613 | Miltenyi Biotec |
|  | CD4 | FITC | REA623 | Miltenyi Biotec |
|  | CD14 | FITC | REA599 | Miltenyi Biotec |
|  | CD19 | FITC | REA675 | Miltenyi Biotec |
| NK cell general markers | CD16 | PerCP-Vio700 | REA423 | Miltenyi Biotec |
|  | CD56 | BV786 | NCAM16.2 | BD Biosciences |
| NK maturation marker | CD57 | BV605 | QA17A04 | Biolegend |
| NK cell subpopulation and inhibitory marker | NKG2A* | APC-R700 | REA110 | Miltenyi Biotec |
| NK cell subpopulation and activated markers | NKG2C | PECP-Vio770 | REA205 | Miltenyi Biotec |
|  | NKG2D | BV711 | 1D11 | BD Biosciences |
|  | NKp30 | PE | REA823 | Miltenyi Biotec |
|  | NKp46** | APC-R700 | REA808 | Miltenyi Biotec |
|  | CD3ζ** | APC | 6B10.2 | eBiosciences |
| NK cell activated markers | CD69* | PE-Vio615 | REA824 | Miltenyi Biotec |
|  | CD107a | BV421 | H4A3 | Biolegend |
|  | IFNγ* | APC | B27 | Biolegend |
| NK cell exhausted markers | Tim-3 | PE-Cy5 | F38-2E2 | Biolegend |
|  | TIGIT** | PE-Vio615 | REA1004 | Miltenyi Biotec |

**Flow cytometry panel 1*

***Flow cytometry panel 2*
