# Supplementary figures and images for: NK Count and Natural Cytotoxicity in Immune Nonresponders Versus Responders Living With HIV
Source: J Med Virol. 2025 Jan 27;97(2):e70170. doi: 10.1002/jmv.70170 (PMC11771562; doi:10.1002/jmv.70170)

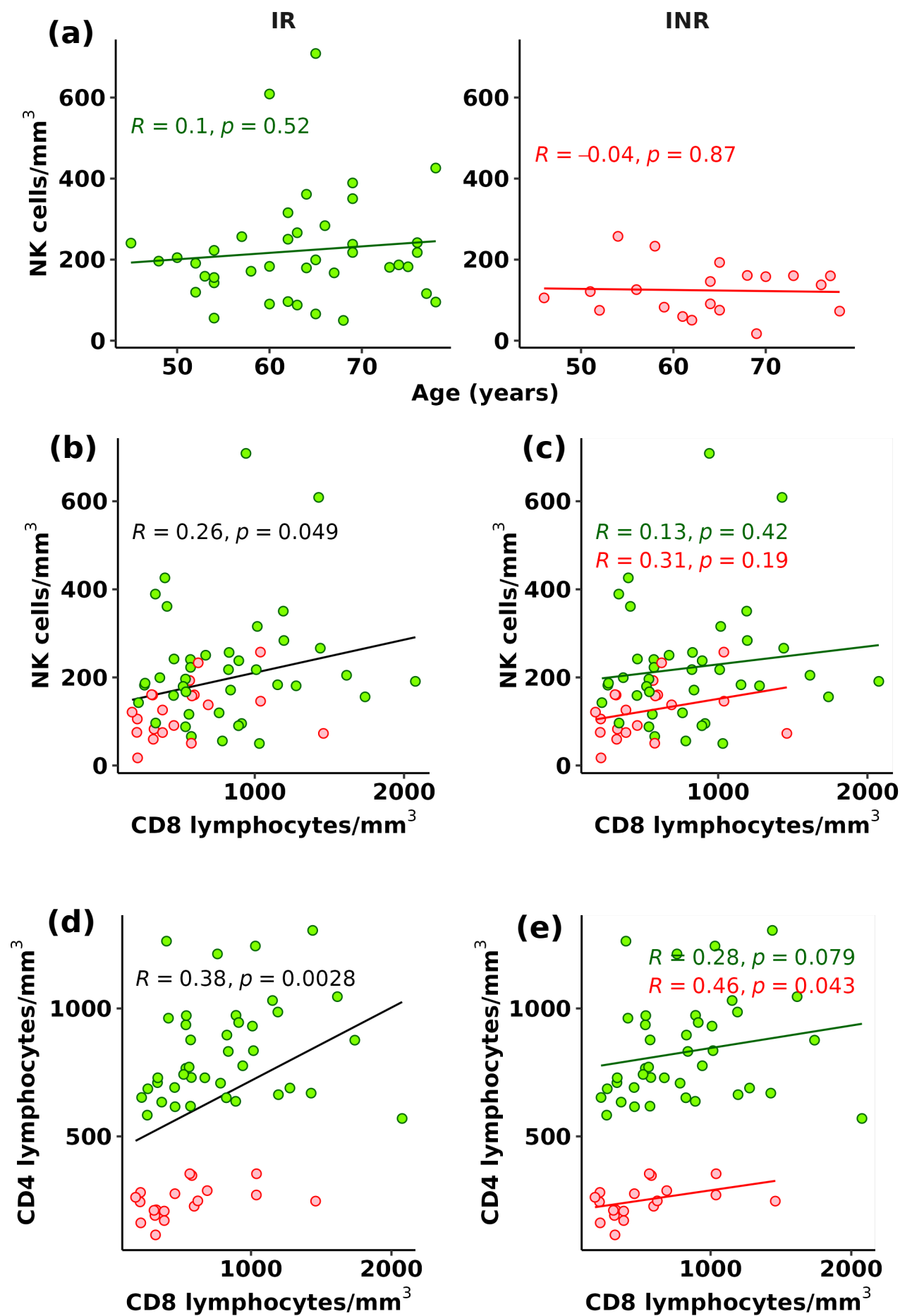

Supplementary figure 1

**(f)**

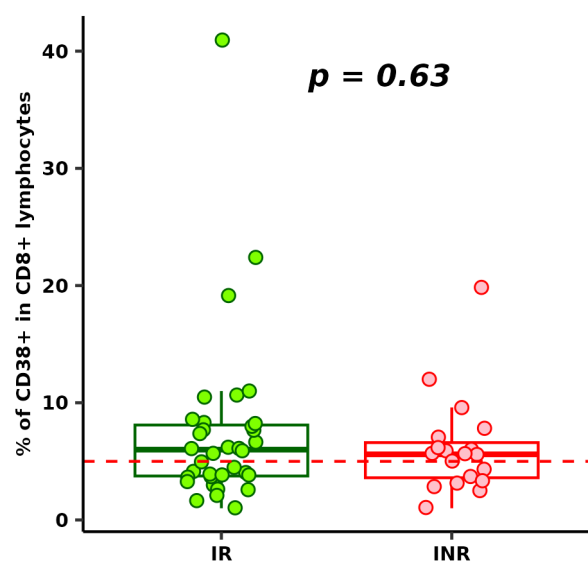

Supplement: Supplementary file 2 — Supplementary Figure 1: (A) Correlation between age and NK count if IR (green) and INR (red) groups. (B, C) Correlation between CD8 counts and NK counts in all enrolled individuals (B) and within each group (C). (D, E) Correlation between CD8 counts and CD4 counts in all enrolled individuals (D) and within each group (E). (F) Percentages of CD8+CD38+ lymphocytes among CD8+ cells in both IR (green) and INR (red) groups. Dashed horizontal red line indicates 5% cut‐off. P‐value computed by Wilcoxon's rank sum test is indicated on the graph. [file JMV-97-e70170-s004.pdf]

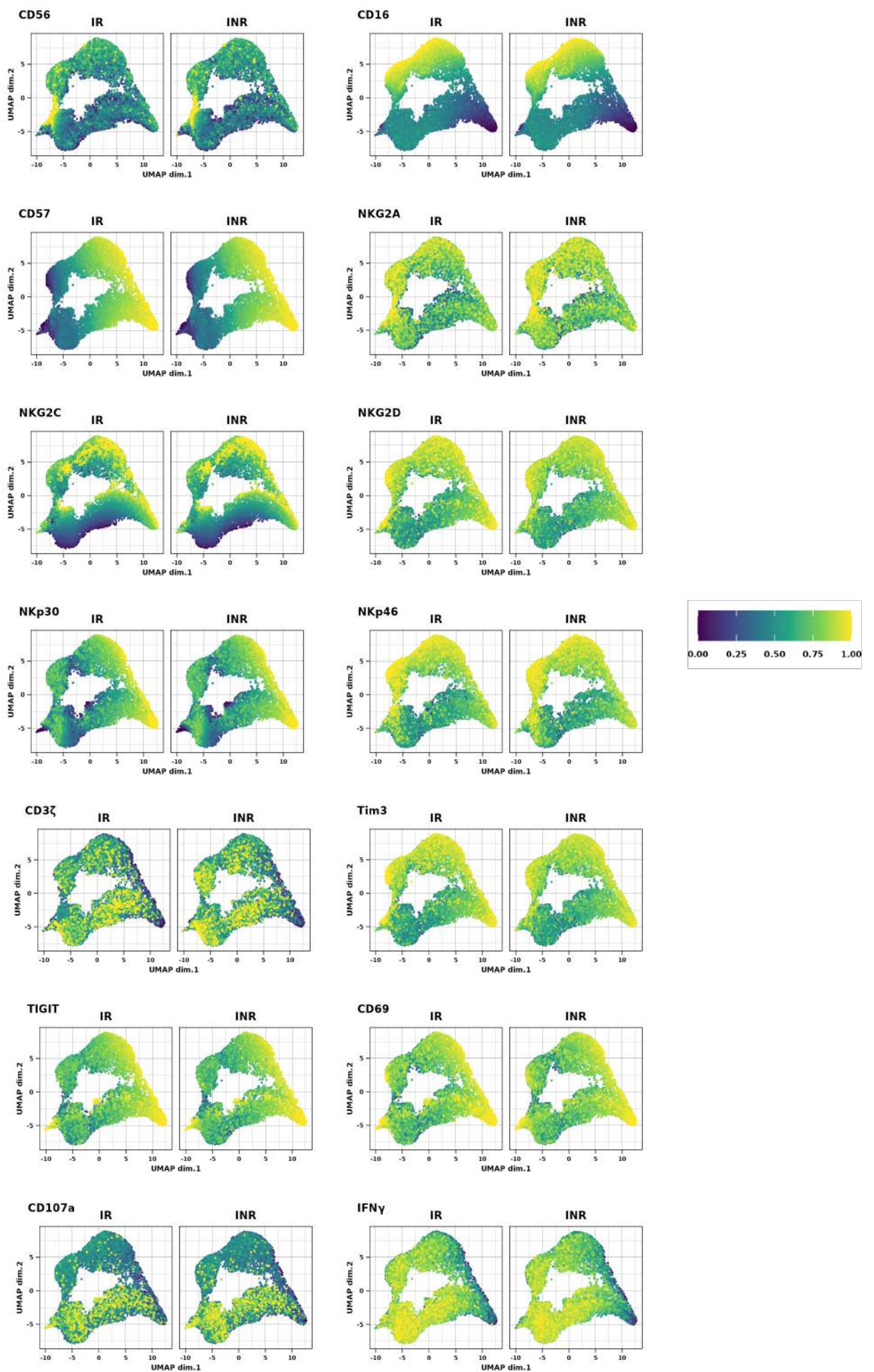

**Supplementary figure 2**

Supplement: Supplementary file 3 — Supplementary Figure 2: UMAP graphs depicting marker expression levels in NK cell population in IR and INR. [file JMV-97-e70170-s002.pdf]

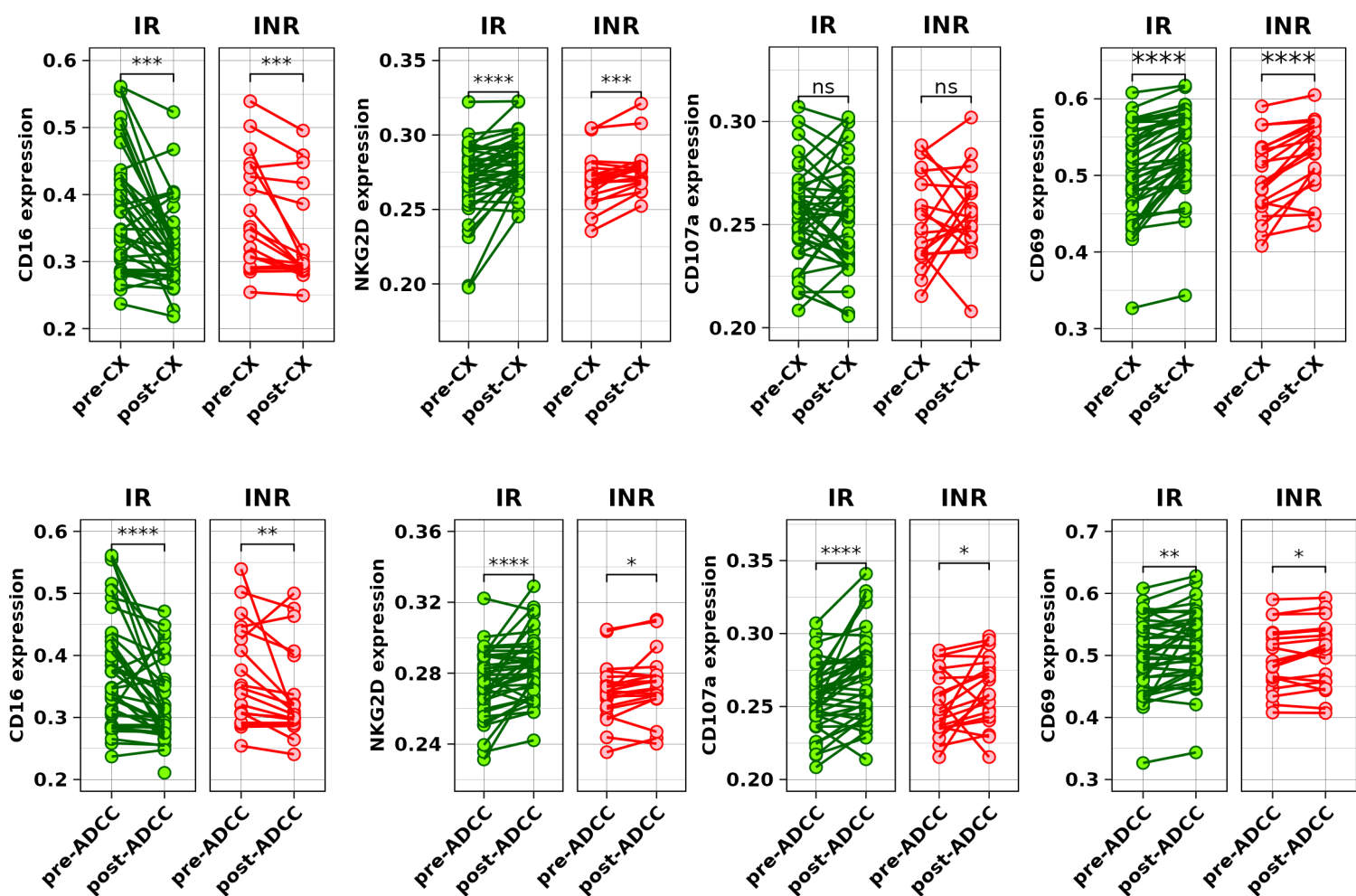

**Supplementary figure 3**

Supplement: Supplementary file 4 — Supplementary Figure 3: Marker expressions before and after cytotoxicity (CX) or ADCC in IR (green circle) and INR (red circle). P‐values of Wilcoxon's signed rank test are indicated on corresponding graphs: ns, p > 0.05, * p < 0.05, ** p < 0.01, *** p < 0.001. [file JMV-97-e70170-s005.pdf]

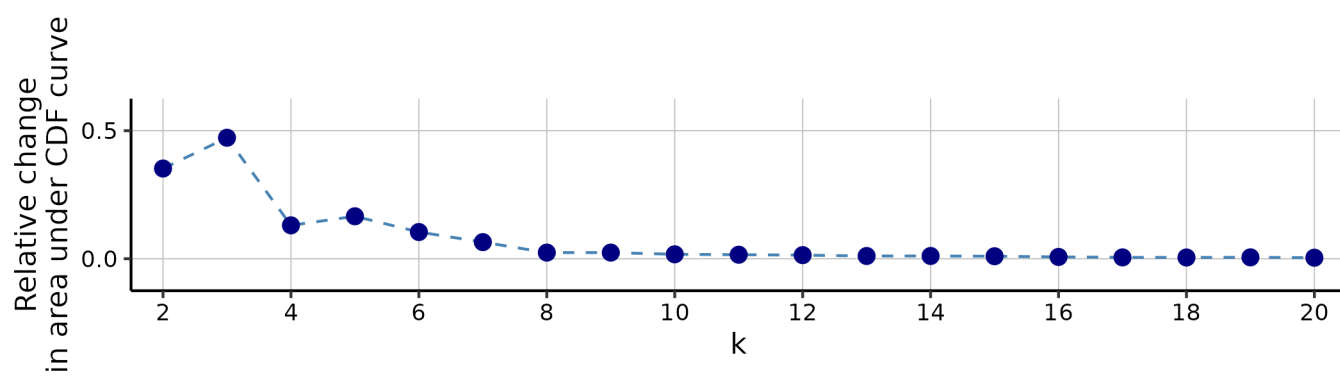

**Supplementary figure 4**

Supplement: Supplementary file 5 — Supplementary Figure 4: Delta area graph from CATALYST package for metaclusterization process. [file JMV-97-e70170-s003.pdf]

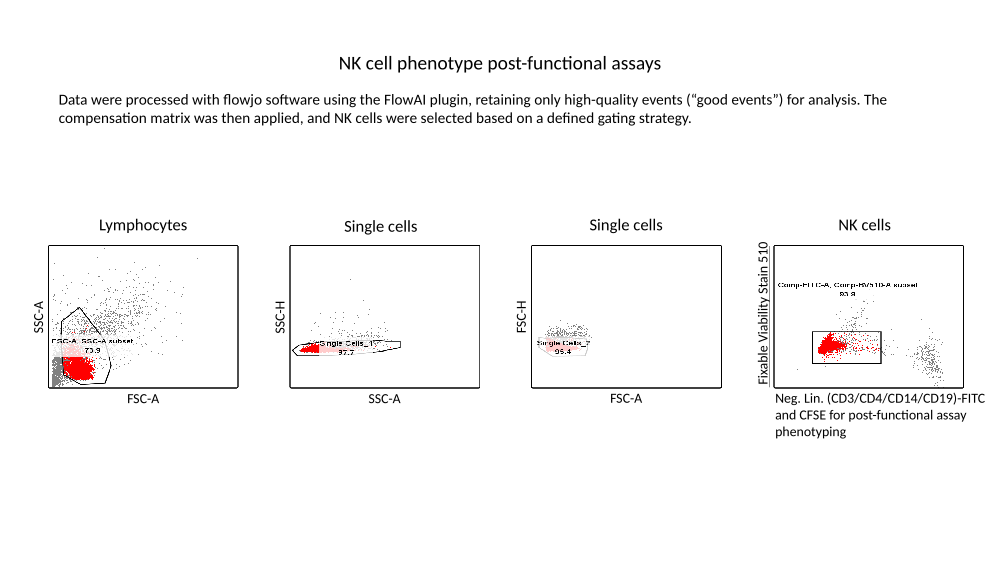

Supplement: Supplementary file 6 — Supplementary Figure 5: Flow cytometry gating strategy on Flowjo. [file JMV-97-e70170-s006.tiff]
